# Correction: Task-based explanation for genre effects: Evidence from a dependency treebank

**DOI:** 10.1371/journal.pone.0324554

**Published:** 2025-05-12

**Authors:** The PLOS One Staff

**Notice of Republication**

This article was republished on March 28, 2025, to correct an error in the second author’s name. The correct name is: Jingyang Jiang. The correct citation is: Wang Y, Jiang J (2023) Task-based explanation for genre effects: Evidence from a dependency treebank. PLoS ONE 18(8): e0290381. https://doi.org/10.1371/journal.pone.0290381

The copyright statement for this article has also been updated to include the corrected author’s name.

## Supporting information

S1 File Originally published, uncorrected article. (PDF)

S2 File Republished, corrected article. (PDF)
