# Supplementary material for: Correction: Task-based explanation for genre effects: Evidence from a dependency treebank
Source: PLoS One. 2025 May 12;20(5):e0324554. doi: 10.1371/journal.pone.0324554 (PMC12068559; doi:10.1371/journal.pone.0324554)
Supplement: S1 File — (PDF) [file pone.0324554.s001.pdf]

RESEARCH ARTICLE

Task-based explanation for genre effects: Evidence from a dependency treebank

Yixin Wang, Jiang Jingyang *

Department of Linguistics, Zhejiang University, Hangzhou, Zhejiang, China

* jjy_203@yeah.net

Check for updates

Abstract

In task-based second language (L2) writing research, genre effects on linguistic features are usually explained by either task complexity hypothesis or differences in communicative demands. The basic distinction between the two explanations is determined by whether cognitive factors are at work. To date, the actual causes for L2 learners' different linguistic features in different genres are still unclear. Aiming at providing empirical evidence for explaining the mechanism of genre effects, this investigation uses dependency-grammar-based measures to examine the role of cognitive factors in L2 argumentative, narrative, and descriptive writings. A total of 540 compositions from three different proficiency groups of English as a foreign language learners were collected, and their mean dependency distances and their distributions of dependency distance were calculated. It was found that in all proficiency groups of compositions, dependency distance distributions of five types showed significant differences between genres. Since dependency distance reflects cognitive load, those five dependency types were able to show that cognitive factors are at play in the writing process. Among the five types, the phrasal dependency relation types could reveal genre effects regardless of learners' language proficiency, and clausal dependency relation types might pinpoint learners' threshold of perceiving task complexity. The findings suggest that genre effects on linguistic features in L2 writings may result from different cognitive demand imposed by writing tasks with different genres, and genre effect may exhibit variation among different proficiency groups.

OPEN ACCESS

Citation: Wang Y, Jingyang J (2023) Task-based explanation for genre effects: Evidence from a dependency treebank. PLoS ONE 18(8): e0290381. https://doi.org/10.1371/journal.pone.0290381

Editor: Heba El-Fiqi, University of New South Wales Canberra, AUSTRALIA

Received: November 29, 2022

Accepted: August 8, 2023

Published: August 23, 2023

Copyright: © 2023 Wang, Jingyang. This is an open access article distributed under the terms of the Creative Commons Attribution License, which permits unrestricted use, distribution, and reproduction in any medium, provided the original author and source are credited.

Data Availability Statement: All relevant data are within the paper and its Supporting information files.

Funding: H. L. 22JJD740018 The MOE (Ministry of Education) Project of Key Research Institute of Humanities and Social Sciences at Universities in China http://www.moe.gov.cn/s78/A13/ The funders had no role in study design, data collection and analysis, decision to publish, or preparation of the manuscript.

Competing interests: The authors have declared that no competing interests exist.

Introduction

Genres are socially constructed language practices that serve specific social purposes [1], such as inviting, arguing, and persuading. Over the years, genre in pedagogical discourses has received considerable attention as it has effects on learners' writing performance. A number of studies have investigated differences in linguistic features caused by writing tasks of different genres, which is helpful for students to discern generic features in writing, and beneficial for teachers to conduct assessments. It has been found that genres in different writing tasks influence syntactic complexity [2–4], accuracy [5, 6], fluency [6], and lexical complexity [2]. Generally speaking, differences in linguistic features caused by task types of different genres, i.e.,

genre effects in this study, have been interpreted from two perspectives. One is the task complexity hypothesis, which includes the Limited Attentional Capacity Model [7] and the Cognition Hypothesis [8–10]. In task-based studies, genre has been operationalized as a task complexity variable, based on the assumption that argumentative tasks that involve logical causal reasoning would be more cognitively demanding to L2 learners than narrative tasks [11]. The other is a functional explanation that suggests genre effects on linguistic performance derive from communicative functions [12] with no cognitive factors at work. From this perspective, task type, instead of task complexity, plays the major part in affecting linguistic performance.

However, there is a notable paucity of empirical research that focuses on providing solid support for explaining the mechanism of genre effects. Since genre is obviously important to L2 writing research and it also has implications for L2 writing theory and L2 writing pedagogy and assessment [6], it is necessary to probe into the mechanism of genre effects. To fill this knowledge gap, this study plans to seek empirical evidence so as to find out the nature of genre effects in L2 writing tasks. Genres, in the writing tasks of this study, will be classified based on pedagogical purposes [13], and will be used as a broader term for the types of writing tasks that students in school education are most frequently exposed to (e.g., "description", "narration" and "argumentation").

Genre effects under the task complexity hypothesis

Task complexity refers to "the result of attentional, memory, reasoning, and other information processing demands imposed by the structure of the task to the language learner" [8]. Despite the fact that it is initially raised for oral production studies, task complexity has been tested in a number of writing studies [14–16].

In order to make predictions about the relationship between cognitive task complexity and students' linguistic performance, two frameworks have been raised. They are Skehan and Foster's Limited Attentional Capacity Model (also known as the Trade-Off Hypothesis) [7] and Cognition Hypothesis (also known as the Triadic Componential Framework) by Robinson [8–10]. These two models have hypothesised how task affects learning by imposing task-related variables on learners' cognitive resources. The Limited Attentional Capacity Model is derived from working memory theory [17], and suggests that tasks are cognitively demanding [7] with the limited information processing capacity of L2 learners. In this sense, tasks with higher complexity result in simpler linguistic output. By contrast, the Cognition Hypothesis, which is based on the processing theories [18], postulates that learners are able to manipulate cognitive resources. According to the Cognition Hypothesis, increasing task complexity simultaneously results in better language performance [9, 19].

The Cognition Hypothesis includes two dimensions, namely the resource-dispersing dimension and the resource-directing dimension. The resource-dispersing dimension argues that task complexity poses procedural demands including "planning", "single task", and "prior knowledge"; and the resource-directing dimension is where task complexity makes conceptual demands on the learner, such as having "here-and-now" and "reasoning demands" [9]. Following the resource-directing dimension, tasks of different genres are hypothesized to have distinct cognitive demands, leading to diverse linguistic output. For instance, Zhan *et al.* [14] focused on an argumentative writing task and a narrative writing task, with control of "reasoning" demand. Their study revealed that the length of unit was positively affected by means of increasing task complexity, and lexical complexity and accuracy were negatively affected. Similar results have also been reported from Ishikawa [20], Ruiz-Funes [21], and Rahimi [22], that increasing task complexity generally elicited more complex syntactic use. However, Cho [23]

controlled the "Few elements" factor by using a programme application and recommendation, and found that simple tasks produced more words per unit, but no difference existed in accuracy and fluency. The controversy necessitates further investigation into task complexity. Moreover, as genre differences involve multiple cognitive factors, a comparison of various task complexity factors is needed. Alexopoulou *et al.* [24] made their investigation based on "numbers of elements", "here and how", "reasoning" and "perspective taking" factors in their research design by adopting two professional writing tasks, two descriptive writing tasks and two narrative writing tasks. Their findings demonstrated that, although not consistently, more cognitively demanding tasks elicited linguistically more complex output both in terms of syntax and lexicon, with little impact on accuracy. Their study demonstrated the significance of task-based L2 writing research across genres by employing three different genre writing tasks with six task design features. However, their design relied on an existing corpus, and offered limited maneuverability in task complexity. To establish a clearer relationship between genre effect and cognitive factors, our aim is to investigate tasks with sequenced complexity.

Taken together, different task complexity caused by different genres has uncertain effects on L2 writing performance. In addition, previous task-based studies have mainly focused on exploring whether their results correspond to the predictions of the task complexity frameworks, instead of understanding the motivation of written language differences [11]. Therefore, what we need is new empirical research to enhance our understanding of the nexus between task complexity and genre effects.

Genre effects under the functional based approach

The other possible explanation of genre effects is based on a socio-cultural view, and starts from the functional-based approach. That is, linguistic differences among genres result from communicative functions instead of different cognitive demands. Along this explanation, it is suggested that genres have communicative requirements in different contexts, and linguistic features are associated with those contexts [12]. For example, the linguistic features for opening a newspaper story include past tense verbs and passive voice verbs [12]. From this perspective, task types, instead of task complexity, plays the major part in affecting linguistic performance.

Following this assumption, Beers and Nagy [25] pointed out that writing high quality texts in different genres may involve acquiring productive control over genre-specific syntactic structures that are tied to the communicative goals of writing. They identified that the relationships between syntactic complexity and text quality were dependent both on the genre of the text, and the measure of syntactic complexity. Following Beers and Nagy [25], Lu [4] used his syntactic complexity analyser to examine syntactic features in narrative writings and argumentative writings. Based on his study, syntactic complexity differed in all indices except T-units per sentence between the two genres.

In recent years, a few studies subscribed to the belief of functional based explanation of genre effects through eliminating cognition-related factors. Yoon and Polio [6] detected the absence of cognitive demand by comparing writings from native English speakers and L2 learners. Their results showed that L2 writings share similar linguistic features with writings of native English speakers and lend support to the functional explanation of genre differences. From their study, argumentative writings had higher length-of-unit complexity, but clausal measures remained identical between genres. Yoon [11] used questionnaires to collect learners' task perception. The result demonstrated that the students did not perceive different genres as imposing significantly different levels of complexity and difficulty. It is therefore reported that the connection between cognitive task complexity and linguistic complexity is

weak, and genre-specific communicative functions was the key motivation for syntactic changes.

In summary, genre effects on writing performance are diverse, and the mechanism of genre effects is still lacking. What is also worthy of mention is that studies that investigated the motivation of genre effect mainly focused on learners of certain proficiency groups, unaware of the latent influence of students' changing proficiency along the development [3]. Given these gaps, we set off to examine the cause of genre effects by exploring whether cognitive factors are at work among different genres. To fully judge whether cognitive complexity affected students' writings, here we propose a very effective metric based on dependency grammar—dependency distance, to measure the cognitive load.

Detecting cognitive factors: Dependency distance as a metric of cognitive load

To explain the mechanism of genre effects, previous studies have adopted some well-designed approaches. Yoon and Polio [6] compared native speaker' writings with English as a foreign language (EFL) learners' writings to detect cognitive factors. Further, Yoon [11] used questionnaires to investigate students' perception of tasks. Ingenious as the methods were, it would be preferable for us if we can measure cognitive load and analyse linguistic features directly from learners' written texts, because this method is more objective. So in this study, dependency-grammar-based (DG-based) metrics will be used to measure cognitive load [26].

Dependency grammar (DG) assumes that the syntactic structures of a sentence are composed of dependencies between individual words [27, 28]. A syntactic dependency relation comprises three core features: (1) It is a binary relation between two words; (2) It is usually asymmetrical, one of the two words serving as the governor (or head) and the other as the dependent; (3) It is classified according to the scope of general syntactic relations, as conventionally shown by the label at the top of the arc connecting the two words [27, 29, 30]. Fig 1 illustrates the dependency relations of the sentence "I consider him as a nice person." The labelled arc is directed from the governor to the dependent. For example, in Fig 1, the head "consider" and the dependent "I" form the dependency relation type *nsubj* (nominal subject), and the governor "as" and the dependent "person" form the dependency relation type *pobj* (object of a preposition).

In dependency grammar, dependency distance (DD) is one of the key concepts. Dependency distance is "the linear distance between the governor and the dependent" [31, 32]. For example, the dependency distance between the governor "as" (the fourth word of the sentence) and the dependent "person" (the seventh word of the sentence) is three. From the psychological perspective, dependency distance indicates how long the earlier word must be kept "active" in working memory during language processing [33]. That is, longer dependency distance entails heavier cognitive load.

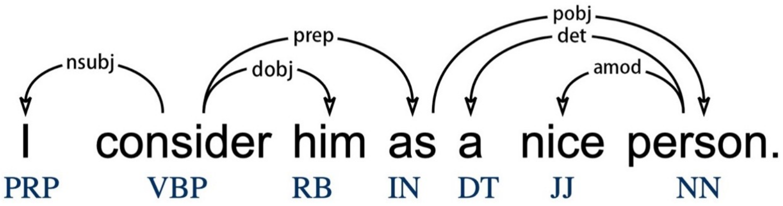

Fig 1. The syntactic dependency relation of one sentence.

https://doi.org/10.1371/journal.pone.0290381.g001

Corpus-based investigations have shown that dependency distance can be held as an important index of memory burden [31, 34, 35]. The greater the dependency distance, the more difficult the syntactic analysis of a sentence [26, 36]. To gauge the cognitive burden of a large text, mean dependency distance (MDD) can be used [37].

The MDD of a text can be calculated using the following formula:

$$MDD\,(the\ text) = \frac{1}{n-s}\sum_{i=1}^{n-s}|DD_i|$$

Here "n" is the number of words in the text and "s" is the total number of sentences in the text. DD_i means the DD of the i-th syntactic link in the text. With dependency distance, it is possible to examine whether differences of cognitive load across a series of genres are significant or not. To be more specific, we will choose different genres and summarise their characteristics along the resource-directing dimension based on the Cognition Hypothesis. Following the assumption of the Cognition Hypothesis, we assume that more complicated tasks lead to heavier cognitive load which is represented by longer dependency distance. If dependency distances show significant differences in the writings of different genres, the explanation that genre effects result from different task complexity will be supported. Based on the introduction, we hereafter raise the following two research questions.

1. From the DG-based metrics, what is the probable explanation of genre effect?
2. To what extent can DG-based metrics reveal genre effects across different educational levels?

Methods

Data

The data were collected from 800 Chinese EFL learners who were in junior high schools, senior high schools and universities (undergraduate and postgraduate) in an eastern province of China to explore genre effects among a variety of learner groups. In this study, all the participants were regular students in the school, and they had the same national English curriculum and used the same set of textbooks. The textbooks and examinations were more difficult for students of a higher educational level. The two high schools were in the same city of Eastern China and were identical in terms of the overall education quality because both belonged to the key schools of the same category. The university was also the key university in China. The teachers of the students who participated in our study were sure that the students' English proficiency of a higher grade was better than that of a lower grade. Since grade is one of the most valid proficiency measures [38], we adopt educational levels to judge the English proficiency levels of the participants. The L1 for the participants was Chinese, and no participants have had the experience of living in an English-speaking country for longer than half a year. The study was approved by the Ethics Committee of the School of International Studies, Zhejiang University. The participants who attained their majority gave informed consent verbally, and we obtained verbal consent from the guardians of the minors included in this study.

Three genres (description, narration and argumentation) were selected in our investigation because they are the most frequently practiced genres in EFL classrooms in China. The topics for descriptive writings were "My father" or "My mother", the topic for narrative writings was "My last weekend", and the topic for argumentative writings was "City life and countryside, which one do you like better?". The evaluation of task complexity followed the Cognition Hypothesis [9, 39]. Table 1 provides an overview of task characteristics of the genres in this

Table 1. Overview of task characteristics.

	Descriptive	Narrative	Argumentative
Number of Elements	Medium-similar	Many-similar	Many-different
Here & now	There & then and Here & now	There & then	There & then and Here & now
Reasoning	No	No	Yes

https://doi.org/10.1371/journal.pone.0290381.t001

study. In brief, the descriptive task was least cognitively demanding and the argumentative task was most cognitively demanding, with narrations in the middle. In order to guarantee the consistency of task complexity, two experienced teachers helped make judgments on the tasks.

To collect those texts, students were given 30 minutes in the classroom to write a composition without referring to other materials. In order to answer the research questions, we randomly selected 540 compositions in total, with 60 for each genre at each level. Table 2 provides an overview of our dataset.

Instruments and measures

The dependency relation tagging of our datasets was first automatically done by Stanford Parser 3.6.0, a syntactically annotated software developed by Stanford University [40]. Stanford Parser provides a total number of 52 dependency relation types, including a variety of linguistic features in the texts, ranging from phrasal features to syntactic features. Although the students had already learned English for years, it was still inevitable for them to make language mistakes, thus increasing the inaccuracy of the program. In addition, despite the fact that Stanford Parser can annotate most of the raw data accurately, there are still quite a few mistakes because the accuracy of the program itself does not reach 100%. To ensure the accuracy and to adapt the raw data to our research, three postgraduate students who are well-acquainted with dependency grammar manually checked the output from the parser. Errors in the students' compositions that affected dependency relations and dependency distances in the sentences were excluded from the statistical analyses in order to gauge cognitive load precisely and accurately. For example, in the sentence "I live in the Hangzhou.", we removed the misused determiner "the" when coding.

Results were saved as spreadsheets (See Fig 2). The distances were extracted from the "Absolute DD (dependency distance)" column.

The measurements include overall mean dependency distance (OMDD), and dependency distance distributions of different dependency relation types. OMDD is calculated by dividing the total dependency distances of a text by the total number of dependency relations of the text [41]. Using OMDD helps us to ascertain whether different genres have significant differences in cognitive load in a broad sense before we probe into specific structures. Meanwhile, all the dependency relation types provided by Stanford Parser are going to be employed, and their dependency distance distributions will be calculated. A prominent advantage of employing dependency relation types is that they are able to represent a wide range of linguistic features

Table 2. Numbers of writings in the L2 writing dataset.

	Descriptive		Narrative		Argumentative	
	Texts	Tokens	Texts	Tokens	Texts	Tokens
Junior high school	60	6201	60	6411	60	6639
Senior high school	60	9732	60	10108	60	9650
University	60	13282	60	14571	60	13947

https://doi.org/10.1371/journal.pone.0290381.t002

Genre	Text Number	Sentence Number	Word Order	Word	POS	Governor Order	Governor	Dependency Relation	DD	Absolute DD
1	1	1	1	The	DT	2	fantasy	det	1	1
1	1	1	2	fantasy	NN	12	been	nsubj	10	10
1	1	1	3	of	IN	2	fantasy	prep	-1	1
1	1	1	4	a	DT	8	life	det	4	4
1	1	1	5	free	JJ	8	life	amod	3	3
1	1	1	6	and	CC	5	free	cc	-1	1
1	1	1	7	healthy	JJ	5	free	conj	-2	2
1	1	1	8	life	NN	3	of	pobj	-5	5
1	1	1	9	in	IN	8	life	prep	-1	1
1	1	1	10	countryside	NN	9	in	pobj	-1	1
1	1	1	11	has	VBZ	12	been	aux	1	1
1	1	1	12	been	VBN	12	been	root	0	0
1	1	1	13	a	DT	15	temptation	det	2	2
1	1	1	14	strong	JJ	15	temptation	amod	1	1
1	1	1	15	temptation	NN	12	been	xcomp	-3	3
1	1	1	16	for	IN	15	temptation	prep	-1	1
1	1	1	17	city	NN	18	dwellers	compound	1	1
1	1	1	18	dwellers	NNS	16	for	pobj	-2	2

Fig 2. Screenshot of the spreadsheet form of the dependency treebank.

https://doi.org/10.1371/journal.pone.0290381.g002

and measure dependency distance at the same time. Based on dependency relation types, we can analyse differences in cognitive demand precisely.

Research hypotheses

This research is intended to find evidence for the explanation of the mechanism of genre effects. From what has been mentioned in the previous section, we assume that if genre effects result from communicative demand, then there will be no difference in cognitive load, and thus dependency distances will not show differences between genres. If it is the different task complexity inherent in different genres that causes genre effects, significant differences in dependency distances will possibly occur in the learners' writings. Such differences may be large enough to influence OMDDs, and may possibly be represented by certain dependency relation types. Meanwhile, educational level is an important factor in allocating cognitive resources as skilled writers would require less effort to manage the attentional resources that activate the linguistic information necessary for writing performance [42]. If all genre effects are caused by task-related cognitive factors, we assume there will be no interactive effect between task complexity and learners' language proficiency. It thus requires us to investigate the OMDDs and the dependency distance distributions of dependency relation types in all proficiency groups, as well as the interactive effects between task complexity and proficiency.

Statistical analyses

Several Q-Q plots of the mean dependency distances of the texts showed that OMDDs in all datasets were normally distributed. So, a two-way analysis of co-variance (Two-way ANCOVA) will be conducted to assess OMDDs. In the analysis, genre and educational level will be set as independent variables and the mean length of sentence will be controlled as the co-variate, since sentence length may have an impact on mean dependency distances [43]. Following this, fine-grained indices will be analysed. Since the distributions of dependency distances follow a power-law distribution [43–45], a generalized linear model (GLM) analysis will be conducted. In the analysis, the link function will be set as "logarithmic". Using GLM will enable us to detect the existence of interactive effects between task complexity and proficiency, as well as make pairwise comparisons among different genres. All statistical analyses will be performed using SPSS, version 26.

Results

The results of the Two-way ANCOVA showed OMDDs in neither genre ($F(2, 59) = 1.517$, p = .220, $\eta_p^2 = .007$) nor educational level ($F(2, 59) = .245$, $p = .783$, $\eta_p^2 = .001$) were significantly different. It was thus necessary to probe into the syntactic structures to see if dependency distance distributions on a micro level can reveal different task complexity.

The distributions of dependency distances of the dependency relation types have been analysed with a general linear model and simple main effect analyses between genres. According to the simple main effect analyses, we discovered that five dependency relation types had significant differences in dependency distributions between genres. The five discriminative dependency relation types are: *advcl* (adverbial clauses), *advmod* (adverbial modifiers), *ccomp* (clausal complements), *dobj* (direct objects), and *nsubj* (nominal subjects), and the results of GLM analysis of the five dependency relation types are listed in Table 3. From the results, none of the discriminative dependency relation types had interactive effect between genre and educational levels.

Table 3. Results of the generalized linear model.

		B	SE	z	*p*
advcl	(Intercept)	1.674	0.062	26.921	< .001
	Level	0.096	0.026	3.662	< .001
	Narrative	-0.621	0.095	-0.651	.515
	Descriptive	0.025	0.096	0.262	.793
	Level*Narrative	-0.340	0.340	-0.850	.396
	Level*Descriptive	-0.072	0.040	-1.802	.071
advmod	(Intercept)	0.550	0.053	10.300	< .001
	Level	0.074	0.022	3.351	< .001
	Narrative	-0.107	0.077	-1.389	.165
	Descriptive	-0.191	0.076	-2.505	.012
	Level*Narrative	0.001	0.031	0.041	.967
	Level*Descriptive	0.052	0.032	1.652	.099
ccomp	(Intercept)	1.080	0.078	13.874	< .001
	Level	0.171	0.032	5.305	< .001
	Narrative	0.156	0.116	1.348	.178
	Descriptive	-0.041	0.120	-0.339	.735
	Level*Narrative	-0.067	0.047	-1.409	.159
	Level*Descriptive	-0.033	0.049	-0.689	.491
dobj	(Intercept)	0.680	0.046	14.796	< .001
	Level	0.032	0.019	1.631	0.103
	Narrative	-0.020	0.066	-0.301	0.763
	Descriptive	-0.130	-.069	-1.874	0.061
	Level*Narrative	0.004	0.028	0.149	.881
	Level*Descriptive	0.023	0.029	0.781	.435
nsubj	(Intercept)	0.479	0.035	13.809	< .001
	Level	0.110	0.151	7.304	< .001
	Narrative	-0.306	0.052	-5.892	< .001
	Descriptive	-0.189	0.051	-3.688	< .001
	Level*Narrative	0.043	0.022	1.921	.055
	Level*Descriptive	-0.007	0.022	-0.295	.768

https://doi.org/10.1371/journal.pone.0290381.t003

Table 4. Results of simple main effect analyses of dependency distance distributions of specific dependency relation types in the junior high school student dataset.

	F	*p*	Argumentative-Narrative	Narrative-Descriptive	Argumentative -Descriptive
advcl	.571	.605			
advmod	2.261	.105	.048		
ccomp	1.287	.278			
dobj	47.687	< .001		.008	.013
nsubj	43.219	< .001	< .001		< .001

https://doi.org/10.1371/journal.pone.0290381.t004

Table 5. Results of simple main effect analyses of dependency distance distributions of specific dependency relation types in the senior high school student dataset.

	F	*p*	Argumentative-Narrative	Narrative-Descriptive	Argumentative -Descriptive
advcl	4.258	.015	.004		.003
advmod	3.198	.041	.007		.004
ccomp	2.788	.063		.028	.008
dobj	8.476	.081			.010
nsubj	14.042	< .001	< .001		< .001

https://doi.org/10.1371/journal.pone.0290381.t005

Tables 4–6 show the results of simple main effect analyses of the aforementioned five discriminative dependency relation types, and their mean dependency distances are visualized in Figs 3 and 4.

Tables 4–6 show that *nsubj* (nominal subject), *dobj* (direct object) and *advmod* (adverbial modifiers) significantly differed between genres in all three proficiency groups. Two other dependency relation types, *advcl* (adverbial clause), *ccomp* (clausal complement) revealed different dependency distances in certain proficiency groups (senior high school and university). Figs 3 and 4 illustrate that the dependency distances had a descending pattern from the more complex task to the less complex task, which is consistent with the task characteristics along the resource-directing dimension in the Cognition Hypothesis.

Discussion

Evidence of task differences in genres

With respect to the first research question 'From the DG-based metrics, what is the probable explanation of genre effect?', the results of the Two-way ANCOVA showed no significant difference in the overall mean dependency distances ($p = .057$) among the three genres. Based on the results, it is possible that different genres have no differences from the cognition aspect in general. The outcome was contrary to the L1 context according to Wang and Liu [46], who reported that mean dependency distances are correlated with genres. The discrepancy requires

Table 6. Results of simple main effect analyses of dependency distance distributions of specific dependency relation types in the university student dataset.

	F	*p*	Argumentative-Narrative	Narrative-Descriptive	Argumentative -Descriptive
advcl	6.614	.001	< .001		< .001
advmod	2.166	.115	.004	.031	
ccomp	1.878	.154			.020
dobj	3.168	.042		.035	.047
nsubj	21.296	< .001	< .001		< .001

https://doi.org/10.1371/journal.pone.0290381.t006

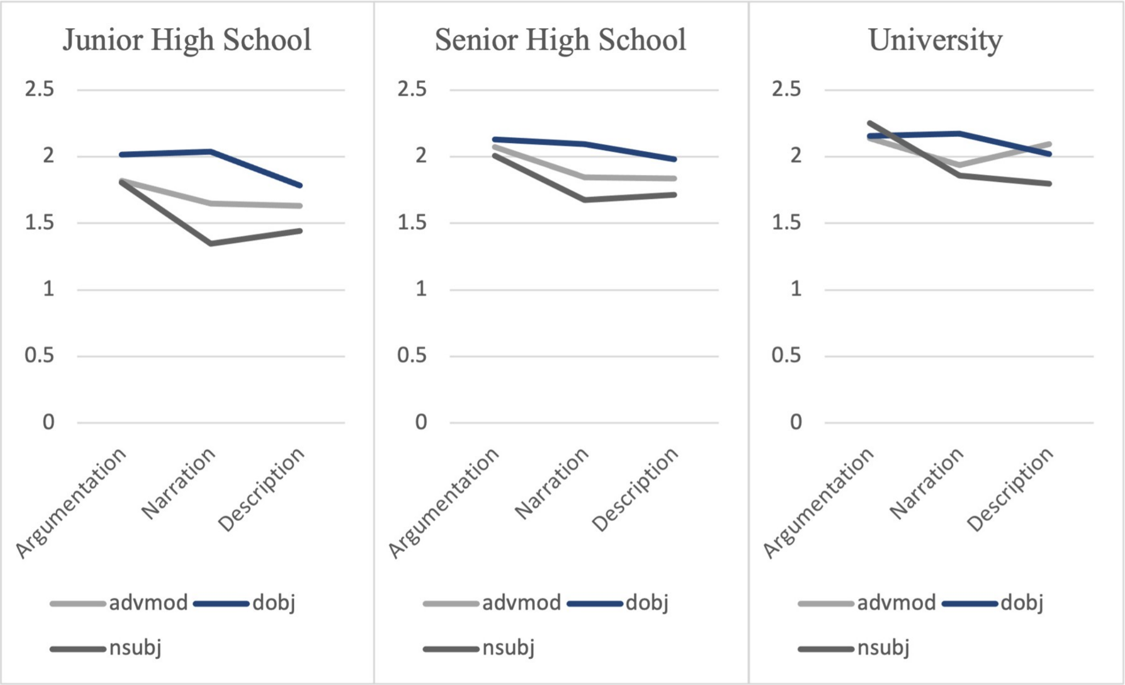

Fig 3. Mean dependency distances of clausal dependency relation types.

https://doi.org/10.1371/journal.pone.0290381.g003

us to probe into more nuanced aspects in L2 writings, that is, dependency distance distributions of dependency relation types.

From the results of simple main effect analyses in the GLM analysis, the dependency relation types which showed significant differences between genres emerged, indicating genres might be cognitively different, and might induce different task complexity.

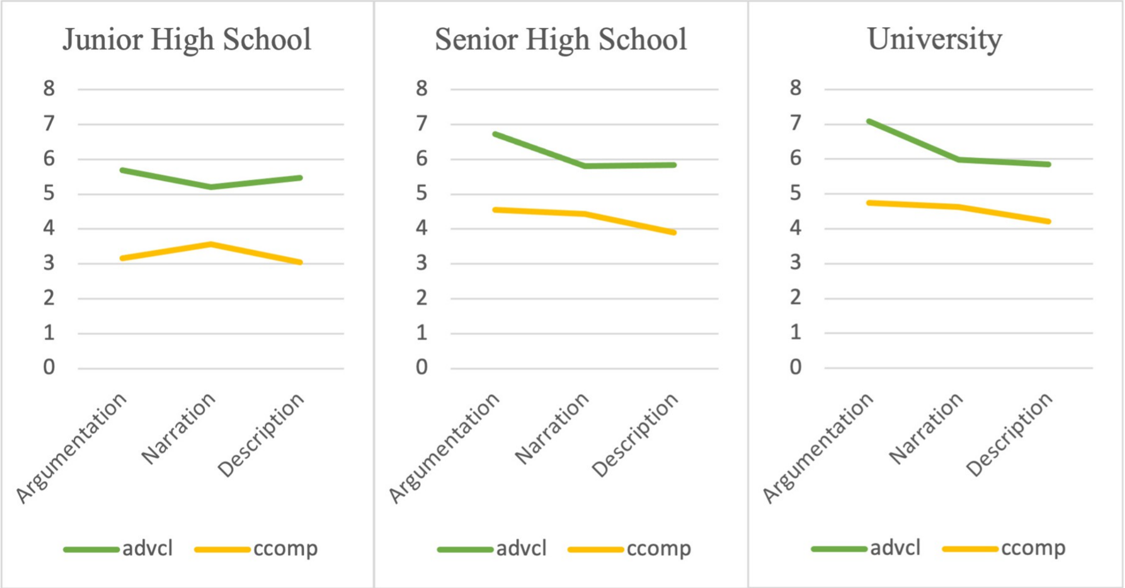

Fig 4. Mean dependency distances of phrasal dependency types.

https://doi.org/10.1371/journal.pone.0290381.g004

Results show that five dependency relation types had significant differences between genres and had insignificant interactive effects with educational levels (*p* values were equal to or smaller than .001). The MDDs of the discriminators appeared the longest in argumentations, and shortest in descriptions. Dependency distances in narrations showed a changing pattern, but had no tendency of being the highest or the lowest.

The existence of discriminative dependency relation types indicates that differences of cognitive load may exist among different genres, and the insignificant interactive effect with institutional levels helped eradicate the potential influence of language proficiency. More importantly, their corresponding mean dependency distances with our research hypothesis along the resource-directing dimension of the Cognition Hypothesis made it clear that genre effects on linguistic features result from the possible differences of cognitive demand inherent in different genres.

Proficiency-free dependency relation types

To answer the second research question "To what extent can DG-based metrics reveal genre effects across different educational levels?", it is necessary to thoroughly investigate the relationship between dependency relations and cognitive factors of writings of different educational levels.

The discriminative dependency relation types can be grouped into two categories according to whether they could differentiate task characteristics at all educational levels. For the dependency relation types that could reveal the differences between tasks independent of language proficiency, we label them as proficiency-free. The dependency relation types in the second category are proficiency-related, which means that they revealed task differences at certain educational levels. The proficiency-free category includes nominal subject, direct object and adverbial modifiers. All of them are phrasal structures.

Nominal subjects had longer dependency distances in argumentative writings, and its MDDs remained identical in narrative and descriptive writings in all proficiency groups. Since argumentative writings have one more cognitive factor (reasoning demand) than narrative writings and descriptive writings, the longer mean dependency distance in argumentative writings might represent the cognitive demand imposed by the "reasoning" factor. In most cases, a nominal subject dependency relation type consists of a verb as the governor, and the nominal subject of the clause as the dependent. The relatively high information density raised the numbers of modifiers. Since the modifiers are frequently added between the governor and the dependent, the dependency distance of this dependency relation type was lengthened. The growing complexity of this phrasal dependency relation type in more complicated tasks shares the result with the study of Zhan *et al.* [14]. In their study, it was discovered that complex writing tasks elicit more complex nominals in the sentences to express their views accurately and clearly. Whilst most of the previous studies conducted experiments with frequency-based indices, we evaluated dependency distances and found supporting evidence from the cognitive perspective. Amongst all the discriminative dependency relation types, nominal subjects were able to reflect differences in cognitive load amongst nearly all genres in our study from all educational levels. Such robustness has been asserted in L1 genre study of Wang and Yan [47], who reported that nominal subjects can effectively measure different genres in L1 writings by employing a quantitative measure to analyze distributions of dependency distances.

Adverbial modifiers, whose dependency distance distributions and mean dependency distances in the three genres showed a similar pattern with nominal subjects. As such, adverbial modifiers might also be sensitive to the "reasoning" factor. Adverbial modifiers have received scant attention in previous studies. Among the available investigations, Alexopoulou *et al.* [24]

affirmed the prediction in their study that narratives will elicit locative adverbs. However, in our research, we found greater dependency distances in adverbial modifiers in argumentative writings instead of word types. Longer dependency distances revealed greater cognitive burden induced by the reasoning demand.

The direct object relation type is composed of a governor (usually a verb) and the direct object of it. Concerning its construction, direct objects are close to nominal subjects, as both of them are formed with a verb and a noun. However, in terms of its role in revealing cognitive factors, direct objects acted differently. The MDDs of direct objects in descriptive writings were lower than those in argumentations and narrations, and turned to be similar between argumentations and narrations. From the task characteristics of our research, the major difference between descriptive writing and the other two writing tasks is the "number of elements" factor (Medium-similar in descriptions and Many-similar in narrations). The lighter cognitive load in descriptive writing tasks was represented by shorter dependency distances of direct objects.

The above three phrasal dependency relation types revealed the property of genre effects regardless of learners' language proficiency, which is consistent with the findings of Kuiken and Vedder [48], who suggested that proficiency was not a major role in syntactic complexity changes. In the meantime, different from Kuiken and Vedder [48], we also found that some of the dependency relation types showed a proficiency-related pattern in terms of dependency distances in three proficiency groups. Such discoveries may help us find unique features of genre effects on a variety of educational levels.

Proficiency-related dependency relation types

Taking a closer look at the five dependency relation types, not all of them could differentiate genres at all educational levels. Dependency distance distributions of two dependency relation types did not differ among genres in the high school dataset. Those discriminative dependency relation types that occurred in certain proficiency groups may get interpretation from the Threshold Hypothesis [49]. The Threshold Hypothesis suggests that it is necessary for L2 writers to achieve a certain level of proficiency before they can do a specific writing task in that language [5]. For adverbial clauses and clausal complements dependency relation types, it was probable that students were not able to perceive task differences on the two linguistic features.

The proficiency-related category includes clausal complement and adverbial clause. A clausal complement dependency relation type is a dependent clause with an internal subject that functions like an object of the verb, or adjective. Clausal complements for nouns are limited to complement clauses with a subset of nouns like "fact" or "report" [40]. From Figs 3 and 4, MDDs of clausal complements showed a clear descending pattern from argumentative writings to descriptive writings in the senior high school dataset and university dataset. In the previous studies that based on frequencies, complement clauses tend to be used by lower proficiency EFL learners, as they frequently use mental verbs [25]. Higher-level learners, by contrast, have the ability to use other syntactic means and constructions for clausal integration [50], hence use fewer clausal complements. However, based on our findings, clausal complements could better discriminate cognitive factors among higher-level EFL learners. With regard to the task characteristics in Table 2, clausal complements might reflect both "numbers of elements" and "reasoning" demand. As for junior high school students, the insignificant difference may be due to the Threshold Hypothesis, suggesting that junior high school students who participated in our research were not able to perceive the differences of cognitive complexity between the genres.

Adverbial clauses are found to be more frequently used in L1 English non-narrative writings among native speakers in previous studies [e.g., 51], and more frequently appear among higher-level EFL learners [50]. Among the research findings of ours, MDDs of adverbial clauses were higher in argumentative writings than in other two genres, while the difference of MDDs of adverbial clauses in narrations and descriptions was insignificant in senior high school and university datasets. Hence, adverbial clauses seemed to be effective in reflecting the "reasoning" factor, and part of the "number of elements" factor on the two relevant educational levels. In the same vein with clausal complements, junior high school students might lack the ability to respond to this cognitive factor.

Contradictory to some of the previous studies, in which task complexity had no significant effect on subordination and coordination [14], we found two dependency relation types related with subordinate clauses (adverbial clauses and clausal complements) were able to differentiate cognitive factors. In addition to the view that more complex tasks elicit more subordinate clauses, our results demonstrated that the cognitive demands behind the clauses are different. The dependency relation types with longer dependency distances represented heavier cognitive load on writers from tasks.

Generally speaking, from the results of GLM and simple main effect analyses, a number of dependency relation types that met our research hypothesis emerged, indicating genre effects may result from different task complexity inherent in different genres. Adverbial modifiers, direct objects and nominal subjects stay sensitive to cognitive factors at all educational levels. Among them, the nominal subject dependency relation type is the most robust one, as it is able to reflect task complexity differences from genres across all educational levels. Clausal complements and adverbial clauses reveal the developmental trait of genre effects. Those dependency relation types offered a reference to future DG-based L2 writing studies.

Conclusions and implications

This study used self-built dependency treebank to examine the mechanism of genre effects in L2 writing tasks. As a metric of cognitive load, dependency distance revealed the nexus between linguistic performance and cognitive load of the writing tasks. Through analysing mean dependency distances and distributions of dependency distance on a variety of dependency relation types, we found dependency distances of certain dependency structures in Chinese EFL learners' writings varied with different task complexity along the resource-directing dimension in the Cognition Hypothesis [8–10]. In the meantime, there was no interactive effect between task complexity and proficiency, indicating genre effects from task complexity consistently affected L2 writings regardless of learners' language proficiency and the ability to manage cognitive load.

Stepping further, we found a series of dependency relation types whose dependency distance distributions might reflect cognitive factors. Nominal subjects and adverbial modifiers are possible discriminators in terms of reasoning demand on all educational levels. Direct object shows the differences of the "numbers of elements" factor on all educational levels. When learners enter senior high school, clausal complements may reveal the existence of the "reasoning demand" factor, and "numbers of elements" factor. For further studies on task and L2 writing, those dependency relation types can be taken into consideration to track the development of students' language proficiency. The detailed discoveries may shed light on L2 writing research, as well as providing guidance for teachers to design teaching syllabus corresponding to students' development of language proficiency.

However, this study has some limitations. Although dependency distance is effective in explaining syntactic differences, it fails to analyse lexical features. Since lexical complexity is

another important dimension in language performance assessment, proper methods need to be applied in future studies. Additionally, writings of three genres on the same educational levels were completed by different participants. Future studies may collect compositions of different genres from the same group of students.

Supporting information

S1 File. Minimal data set of the research.
(XLSX)

Author Contributions

Conceptualization: Jiang Jingyang.

Data curation: Jiang Jingyang.

Formal analysis: Yixin Wang.

Funding acquisition: Jiang Jingyang.

Investigation: Yixin Wang.

Methodology: Yixin Wang.

Project administration: Jiang Jingyang.

Resources: Jiang Jingyang.

Software: Yixin Wang.

Supervision: Jiang Jingyang.

Validation: Jiang Jingyang.

Visualization: Yixin Wang.

Writing – original draft: Yixin Wang.

Writing – review & editing: Jiang Jingyang.

References

1. Halliday MAK, Hasan R. Language, context and text: Aspects of language in social-semiotic perspective. Geelong, Victoria: Deakin University Press; 1985.
2. Berman RA, Nir-sagiv B. Comparing Narrative and Expository Text Construction Across Adolescence: A Developmental Paradox. Discourse Processes. 2007; 43(2):79–120.
3. Bi P. Revisiting genre effects on linguistic features of L2 writing: A usage-based perspective. International Journal of Applied Linguistics. 2020; 30(3):429–44.
4. Lu X. A Corpus-Based Evaluation of Syntactic Complexity Measures as Indices of College-Level ESL Writers' Language Development. TESOL Quarterly. 2011; 45(1):36–62.
5. Kuiken F, Mos M, Vedder I. Cognitive task complexity and second language writing performance. In: Foster-Cohen S, García Mayo MdP, Cenoz J, editors. Eurosla Yearbook. 5. Amsterdam: John Benjamins; 2005. p. 195–222.
6. Yoon H-J, Polio C. The Linguistic Development of Students of English as a Second Language in Two Written Genres. TESOL Quarterly. 2017; 51(2):275–301.
7. Skehan P, Foster P. Cognition and second language instruction. In: Robinson P, editor. Cognition and tasks. Cambridge: Cambridge University Press; 2001. p. 183–205.
8. Robinson P. Task complexity, task difficulty, and task production: Exploring interactions in a componential framework. Applied Linguistics. 2001; 22(1):27–57.

9. Robinson P. Cognitive complexity and task sequencing: Studies in a componential framework for second language task design. International Review of Applied Linguistics in Language Teaching. 2005; 43 (1):1–32.
10. Robinson P. Task complexity, cognitive resources, and syllabus design: A triadic framework for examining task influences on SLA. In: Robinson P, editor. Cognition and second language instruction. Cambridge: Cambridge University Press; 2001. p. 287–318.
11. Yoon H-J. Challenging the connection between task perceptions and language use in L2 writing: Genre, cognitive task complexity, and linguistic complexity. Journal of Second Language Writing. 2021; 54:100857.
12. Biber D, Conrad S. Register, genre, and style. Cambridge, UK: Cambridge University Press; 2009.
13. Bhatia VK. Worlds of written discourse: A genre-based view. London: Continuum; 2004.
14. Zhan J, Sun Q, Zhang LJ. Effects of manipulating writing task complexity on learners' performance in completing vocabulary and syntactic tasks. Language Teaching Research. 2021(1):1–22.
15. Jeong H. Narrative and expository genre effects on students, raters, and performance criteria. Assessing Writing. 2017; 31:113–25.
16. Kuiken F, Vedder I. Task complexity and linguistic performance in l2 writing and speaking: The effect of mode. In: Robinson P, editor. Second language task complexity: Researching the cognition hypothesis of language learning and performance. Philadelphia: John Benjamins; 2011. p. 91–104.
17. Carter R. Mapping The Mind. Berkeley: University of California Press; 1998.
18. Schmidt R. Attention. Cognition and second language instruction. Cambridge: Cambridge University Press; 2001. p. 3–32.
19. Robinson P. Second language task complexity, the Cognition Hypothesis, language learn- ing, and performance. In: Robinson P, editor. Second language task complexity: Researching the cognition hypothesis of language learning and performance. Amsterdam: Benjamins; 2011. p. 3–38.
20. Ishikawa T. The effect of manipulating task complexity along the [+/−Here-and-Now] dimension on L2 written narrative discourse. In: García Mayo MdP, editor. Investigating Tasks in Formal Language Learning. Clevedon: Multilingual Matters; 2006. p. 136–56.
21. Ruiz-Funes M. Exploring the potential of second/foreign language writing for language learning: The effects of task factors and learner variables. Journal of Second Language Writing. 2015; 28:1–19.
22. Rahimi M. Effects of increasing the degree of reasoning and the number of elements on L2 argumentative writing. Language Teaching Research. 2018; 23:633–54.
23. Cho M. Task design features and learner variables in task performance and task experience. Honolulu: University of Hawai'iat Mānoa; 2015.
24. Alexopoulou T, Michel M, Murakami A. Task Effects on Linguistic Complexity and Accuracy: A Large-Scale Learner Corpus Analysis Employing Natural Language Processing Techniques. Language Learning. 2017; 67(1):180–208.
25. Beers SF, Nagy WE. Syntactic complexity as a predictor of adolescent writing quality: Which measures? Which genre? Reading and Writing. 2009; 22(2):185–200.
26. Liu H. Dependency Distance as a Metric of Language Comprehension Difficulty. Journal of Cognitive Science. 2008; 9(2):159–91.
27. Hudson R. Language networks: The new word grammar. Oxford: Oxford University Press; 2007.
28. Nivre J. Inductive dependency parsing. Heidelberg: Springer; 2006.
29. Tesnière L. Eléments de syntaxe structurale. Paris: Klincksieck; 1959.
30. Hudson R. An English word grammar. Oxford: Basil Blackwell; 1990.
31. Hudson R. Measuring syntactic difficulty1995 2021 Oct 8. https://dickhudson.com/wp-content/uploads/2013/07/Difficulty.pdf.
32. Heringer HJ, Bruno S, Rainer W. Syntax: Fragen, Lösungen, Alternativen. Munich: Wilhelm Fink Verlag; 1980.
33. Hudson R. The psychological reality of syntactic dependency relations. MTT; Paris 2003.
34. Liu H, Xu C, Liang J. Dependency distance: A new perspective on syntactic patterns in natural languages. Physics of Life Reviews. 2017; 21:171–93. https://doi.org/10.1016/j.plrev.2017.03.002 PMID: 28624589
35. Futrell R, Mahowald K, Gibson E. Large-scale Evidence of Dependency Length Minimization in 37 Languages. Proceedings of the National Academy of Sciences of the United States of America. 2015; 112(33):10336–41. https://doi.org/10.1073/pnas.1502134112 PMID: 26240370
36. Gibson E. Constraints on sentence comprehension. Trends In Cognitive Science. 1998; 2(7):262–8. https://doi.org/10.1016/S1364-6613(98)01187-5 PMID: 21244925

37. Liu H. Dependency grammar: From theory to practice. Beijing: Science Press; 2009.
38. Wolfe-Quintero, Inagaki S, Kim H-Y. Second language development in writing: Measures of fluency, accuracy, and complexity. Honolulu: University of Hawaii Press; 1998.
39. Robinson P, Gilabert R. Task complexity, the Cognition Hypothesis and second language learning and performance. IRAL—International Review of Applied Linguistics in Language Teaching. 2007; 45 (3):161–76.
40. Marneffe M-Cd, Manning CD. Stanford typed dependencies manual. 2008.
41. Ouyang J, Jiang J, Liu H. Dependency distance measures in assessing L2 writing proficiency. Assessing Writing. 2022; 51:100603.
42. Kellogg RT. Competition for Working Memory among Writing Processes. The American Journal of Psychology. 2001; 114(2):175–91. PMID: 11430147
43. Jiang J, Liu H. The effects of sentence length on dependency distance, dependency direction and the implications–Based on a parallel English–Chinese dependency treebank. Language Sciences. 2015; 50:93–104.
44. Ferrer-i-Cancho R. Euclidean distance between syntactically linked words. Physical Review E. 2004; 70(5):056135. https://doi.org/10.1103/PhysRevE.70.056135 PMID: 15600720
45. Liu H. Probability distribution of dependency distance. Glottometrics. 2007; 15:1–12.
46. Wang Y, Liu H. The effects of genre on dependency distance and dependency direction. Language Sciences. 2017; 59:135–47.
47. Wang Y, Yan J. A quantitative analysis on a literary genre essay's syntactic features. Quantitative analysis of dependency structures. Berlin, Boston: De Gruyter Mouton; 2018. p. 295–314.
48. Kuiken F, Vedder I. Syntactic complexity, lexical variation and accuracy as a function of task complexity and proficiency level in L2 writing and speaking. In: Housen A, Kuiken F, Vedder I, editors. Dimensions of L2 Performance and Proficiency. Amsterdam: John Benjamins; 2012. p. 143–69.
49. Cummins J. Cognitive/Academic Language Proficiency, Linguistic Interdependence, the Optimum Age Question and Some Other Matters. Working Papers on Bilingualism, No. 19.; 1979.
50. Jiang J, Bi P, Liu H. Syntactic complexity development in the writings of EFL learners: Insights from a dependency syntactically-annotated corpus. Journal of Second Language Writing. 2019; 46.
51. Scott CM, Windsor J. General Language Performance Measures in Spoken and Written Narrative and Expository Discourse of School-Age Children With Language Learning Disabilities. Journal of Speech, Language, and Hearing Research. 2000; 43:24–339. https://doi.org/10.1044/jslhr.4302.324 PMID: 10757687
